# Wearable technology may assist in reducing jockeys' injuries if integrated into their safety vests: a qualitative study

**DOI:** 10.3389/fspor.2023.1167110

**Published:** 2023-06-21

**Authors:** Lisa Giusti Gestri

**Affiliations:** FBI Lab, Faculty of Art, Design & Architecture (MADA), Monash University, Caulfield East, VIC, Australia

**Keywords:** personal protective equipment, user centered design, wearable sensors, product standards, jockeys’ safety vests, sports medicine, case study

## Abstract

While the term “safety vests” has been used to capture these products to reduce the potential for harm in jockeys under the Personal Protective Equipment (PPE) umbrella, much of the research in this area has focused on factors typically echoing health, well-being, physiological and cognitive function, and performance of horse riders with very little work about examining how its design may reduce the severity of jockeys' injuries. Due to the recent advances in technology and wearable sensors, the author considered a qualitative study focusing on the analysis of a real-life example involving end and co-dependent users in the design development of jockeys' safety vests. This little article offers an overview of the most popular jockeys' injuries, why there is a need for better protection, and also describes how data were collected and present a summary of the key findings to encourage future research in this field, aiming to create a new prototype. High-impact sports may potentially create severe injuries or deaths to athletes: thus, there is a strong faith in the application of wearable sensor data and data science to also enhance jockeys' safety vest performance.

## 1. Introduction

 Sports scientists constantly research newer technologies, data platforms, and any therapies able to help athletes to perform at their best level while minimizing their risk of injuries. Regarding sports teams, many wearable technologies have been recently utilized to quantify their workload (1–3) and to prove a link between it and injury rates (4). According to Vamathevan et al. (5), the opportunity of having open-source repositories of wearable data may simplify collaborations between academics, sports teams, and Personal Protective Equipment (PPE) manufacturers to maximize the health and performance of athletes. Despite the technologies available, it remains still unclear how biomechanical, physiological, and biochemical data relate to injury risk and exactly what the technology may measure to become relevant to athletes' health (6).

For a very long time, researchers of the design of health products have often mentioned the necessity of User Centered Design (UCD) to acknowledge secondary and tertiary users (7–9). Nevertheless, what is missing is a clear definition of the above-mentioned users specifically, that is nuanced to make them the same as primary users in terms of the significance of their input and access role. The term User Experience (UX) was introduced almost three decades ago but since then its original meaning still includes the understanding of users' needs at its core and pursuing what Norman et al. (10) named “joy to use” thus, an interdisciplinary approach is required. However, Berni and Borgianni (11) state that the definition of UX is still vague because of several acceptations with different nuances despite the presence of similarities. As co-design and participatory design processes are increasingly applied across diverse sectors including business, health, government, and education, with resulting success, the value of engaging users and various stakeholders in design processes is further understood and appreciated (12–15). Co-design continues to emerge as a key methodological foundation through which complex, transdisciplinary problems and challenges are addressed: nonetheless, models and design principles for complex, multiple, and diverse stakeholder collaboration are limited (16).

Activity trackers and wearable health devices have significantly grown in popularity and reinforced the idea that the data generated by these devices may help health professionals offer treatments to their patients of better quality. Particularly, to preserve athletes' safety during their sports activities, Personal Protective Equipment (PPE) is the best answer: wearing the appropriate and well-fitted protective equipment, clothing and footwear helps in preventing sports injuries even from a young age (6, 17, 18). Each sport has a diverse nature and the PPE literature arguments ways in which design innovation and advanced materials now available have been utilized to protect athletes from injury (19–21). For instance, investigating the PPE utilized in sports highlighted a lack of product standards' innovation, particularly for the safety vests worn by jockeys because race riding is a dangerous occupation. Horse racing is a popular form of sport among all horse riding branches, but it is associated with a high rate of injuries.

Thoroughbred racing is a popular and major international sport that significantly contributes to the economy of 47 countries around the world (22). Particularly, jockeys are responsible for controlling both their personal and horses' riding performances, while racing at speeds of even more than 60 Kmh^−1^ on race days (23, 24). Contrary to other sports athletes, jockeys are asked to maintain their peak physical condition and low weight on a daily basis because there is no off-season (25). Besides, flat racing jockeys have more race days per season and rides per day than jump racing ones. Globally, horse racing is considered a high-risk and dangerous sport (26, 27): thus, Australia made safety vests compulsory to be worn by jockeys in 1998. Nonetheless, the Australian safety vests currently worn are insufficient in preventing jockeys' injuries: there is a need for ongoing scope for new designs to be introduced to the market especially now that wearable technologies are well diffused into sports and associated with enhanced functionality and design. However, neither incremental nor radical product innovation is actually possible in Australia due to the constraining effects of their product standards, which have been minimally and rarely reviewed over the past decades. It is significant to state that there is no specific literature discussing the design and its combination with technology in the field of jockeys' safety vests.

This study discusses the author's understanding of how safety vests for jockeys may seek aid in wearable technology and advanced materials in guiding their injury minimization, by asking for their standards' revision too via the application of the product design framework to ethnographic research. Because Singh et al. (28) introduced research design in as much as the use of a design method has pivotal relevance due to it needing a very systematic approach the author, consequently, utilized a problem-driven design to accommodate the request for a super-lightweight but still a flexible product to satisfy users' needs. Firmly believing that a design thinking approach may solve problems for humans, and inspired by Brown (29), she referred to it as the heart to match users' needs with what is feasible and convert them into a market opportunity (focusing on safety vests for jockeys). Thus, design thinking is powerful in ensuring continuity in the process of innovation and with the aim of producing an idea and creating a subsequent product; Brown (29) suggests three phases that should be considered during further research in this field: (1) Look at the world (Inspiration); (2) Brainstorm your ideas (Ideation); (3) Execute the vision (Implementation).

Hence, the author focused on the products, their users, and their standards due to the pressures that are restricting their design evolution. The jockeys’ safety vests design has a pivotal role in the evolution and/or implementation of product innovation. Besides, inspired by the definition of UX design as “the perceptions and responses of users that result from their experience of using a product or service” (30, 31), she employed that to identify whole the users that may affect the design development of these products. The literature possesses a decent number of publications debating on the sample size and saturation appropriate for qualitative and even quantitative research (32–36). Therefore, the author preferred the saturation definition as a “matter of degree” and it should be more concerned with reaching the point where it becomes “counter-productive” and that “the new” is discovered does not necessarily add anything to the overall story, model, theory, or framework” (37). Accordingly, she had the perfect opportunity to investigate a product from the users’ point of view because the author also considered how the user-product relationship was influenced by the context within which it was situated while establishing a design framework.

According to Marshall et al. (32), there are three methods to contribute to the sample size applied in qualitative research and their recommendations are:

*“… (a) grounded theory qualitative studies should generally include between 20 and 30 interviews; (b) single case studies should generally contain 15–30 interviews; (c) qualitative researchers should examine the expectations of their intended journal outlets based on history and culture; and (d) replication studies should further examine the impacts of culture and study design. (p.21)”*

Consequently, and in accordance with a substantial number of other publications [e.g. (38–41),], the author was satisfied with an overall number of 20 participants because of the aforementioned points (a), (b) and (c) and because when using the interviews method, saturation is usually achieved in a homogeneous participant group of between 13 and 15 participants. Thus, respecting that and having a sample size that was pertinent to reaching saturation while relying on a various number of factors too (42), the author progressed with a participants group of male and female jockeys, medical professionals, and varying in age and experiences.

## 2. Methods

This study involved users (overall, 20 jockeys and related medical professionals) and their experiences with a specific PPE (safety vests) dedicated to thoroughbred horse riders: however, because no disease was directly involved, it can't be considered an epidemiological one. Nevertheless, the author may have referred to epidemiological publications as another inspiration for this investigation on the design of jockeys' safety vests but from their users' intimate point of view. This case offered the possibility to analyze a product from the users' point of view because the author even took into consideration how this relationship (users—product) was influenced by the context within which it is situated.

In response to the constraints on innovation in the design of jockeys’ safety vests, the author conducted a program of field research to understand jockeys' and medical first responders' perceptions of the safety vests mandated for use by Australian jockeys. The study used a flexible, qualitative research design incorporating semi-structured interviews, a focus group, and observation because each experience with a product has its own individual ecology and mediated dynamic factors. Still, people bring past experiences and expectations to product interaction (43). Hence, the research design was informed by Forlizzi's (44) concept of product ecology, of which she writes:

*“The Product Ecology framework articulates all of the factors that evoke social behavior around products. The factors in the framework can be used in a generative manner to scaffold the selection of design research methods for understanding current experience and generating new products to change that experience …[It] provides an alternative way of understanding the complex physical and social context of use around a product, and a means for suggesting change within the current state of the world”. (p. 18)*

The application of the theoretical framework developed by Forlizzi has supported the understanding of how a product evokes social behavior, offering a path for choosing the appropriate research methods, and expanding the design culture in interaction design that enabled design-centered research. Significantly, products can convey meanings, but these vary for a range of people, at different times, and in various contexts: hence, the research design for this study applied the product ecology with the theoretical framework to increase the sense of *what a product is* and *what it could be* (44). Each user is unique just in associating diverse meanings and feelings with a product due to its everyday use, which leads to a long-established product standard.

### 2.1. Background to horse racing

PPE plays an essential role in preserving athletes' safety when participating in sports: because of the diverse nature of sports and sporting injuries, PPE is a broad product category that may include safety products like helmets, body protectors, gloves, goggles, and mouth guards, with athletes often required to use a combination of equipment to offer full protection (45, 46). The PPE literature discusses how design innovation and advanced materials have been applied to PPE design to offer enhanced protection to athletes, although there is no specific literature on the design of jockeys’ safety vests. Race riding is well-acknowledged as a risky activity: regardless of a jockey's training and skill, it is not possible to prevent a fall. Still, horses are unpredictable animals that have evolved to use agility and speed to escape danger but riding them at speed is yet inherently dangerous (47).

In Australia, horse racing began in 1788 and by the 1830s it was a popular sport. Its popularity prompted the formation of the Victorian Jockeys' Association in March 1858 (48), with a total of 19 jockeys signed up at the first meeting, including Australian-born riders Alice Hawthorn and Stephen Mahon, and the English-born champion Sam Holmes. Australian races include all horses, but they are divided into a grade that matches their abilities and most of them are conducted on a flat surface, which is predominantly grass, or turf as it is most widely known. The Country has two main thoroughbred racing categories: flat racing and jump racing (49). Flat races are run over distances from 800 meters (m) to 3,375 m on mainly turf tracks instead, jump races are run over distances from 3,200 m up to 7,200 m. The author focused on jockeys, which are those who ride horses in horse racing and mainly as a profession: most jockeys are self-employed, attracting a fee for each race they ride and a percentage of any prize money a horse wins in a race. Despite the horses have evolved to use their natural agility and speed as escape mechanisms from dangerous situations, there is still a mere possibility of danger that can prompt a horse to halt abruptly, leap sideways, spin on the spot and gallop away from the potential source of risk. This exposes not only the animals but even the jockeys to danger: although jockeys must wear gloves, helmets, goggles, and safety vests, they are daily exposed to high risk in races and training gallops, accepting that they are likely to experience falls and consequent injuries during their career (50).

The former jockey Brian Rouse has surely described horse racing and its associated risks in a very clear way, comparing them to “like driving a car with no brakes. If you make a mistake, you can't rectify it in one stride” [quoted in (51)]. Despite PPE may not completely cancel all risks from sports, it can minimize injuries and save lives. Therefore, the author focused on Australian jockeys who ride in flat races because every year, more than a third of them are involved in accidents that require medical attention: of those, 40% will have a fall that will prevent them from riding for an average of 5 weeks: even permanent disability is experienced each year by several jockeys (52). Due to the risks that jockeys face, medical staff attend all race meetings to monitor the well-being of riders: during each race, two ambulances follow the track on an adjacent path to provide first aid to jockeys (53–55). Specifically, to ensure racing and training activities are supported by best practice medical services, “raceday doctors travel in the paramedic vehicle behind the race field, supported by 2 Ambulance Transport Attendants or Ambulance Officers and a Registered Critical Care Nurse” (56). With the scope to reduce the severity of jockeys' injuries, in 1995 the Australian Racing Board (ARB) has commissioned a collaborative study among doctors and engineers to investigate how best to protect jockeys (57–60). Following that, in 1998 the ARB made the use of safety vests compulsory and introduced the Australian standard named ARB 1.1998, which was closely aligned and since then used with the European Standard EN 13158.

**Figure 1:**
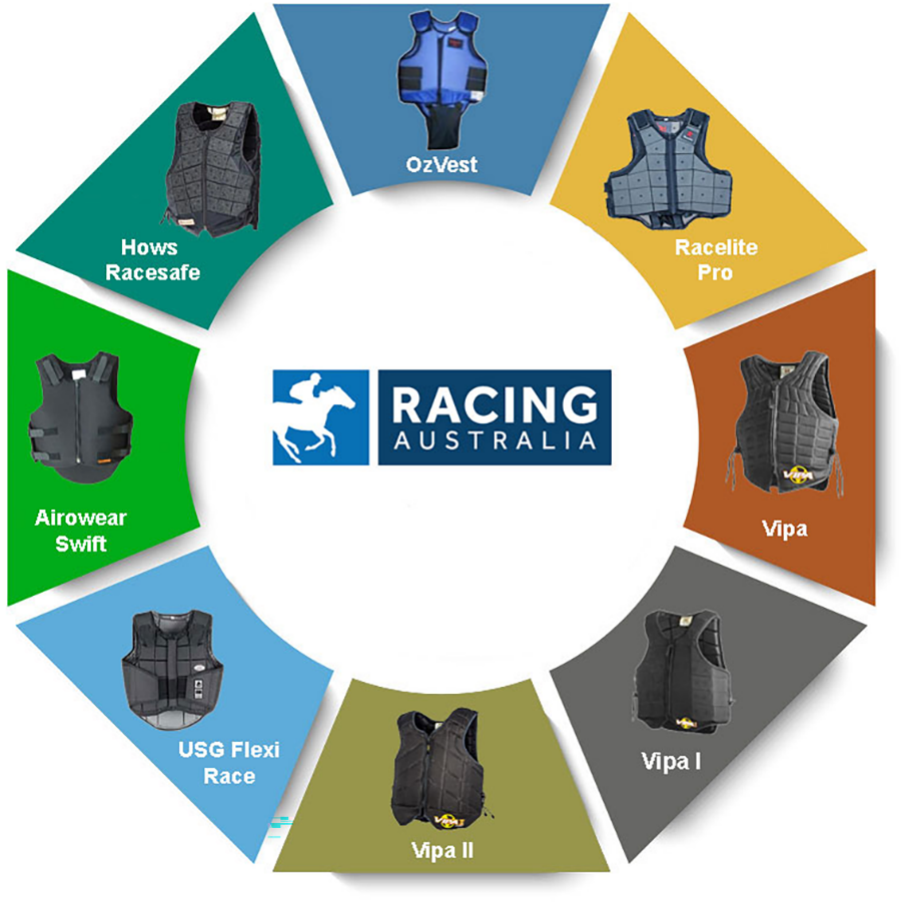
Racing Australia approved jockeys’ safety vests.

Jockeys' safety vests are designed to cover the ribcage, specifically both parts of the abdomen and the back, either during the trackwork or races: they aim to protect jockeys from severe injuries if a fall occurs or a horse's kick is received (27, 61, 62). Since 1998, Australian safety vests for jockeys must comply with the ARB 1.1998 and the EN 13158 standards (63) to accommodate male and female jockeys of varying body types and sizes. These standards determine that safety vests are made of perforated foam strips of varying thickness, covered with mesh polyester, along with adjustable straps or Velcro® sections at the shoulders and waist to keep the vests tight on the jockeys' bodies. However, the lack of innovations due to a scarce revision of the Australian safety vests' standards led to scant design innovation in this field while still exposing users to risks (64, 65). Racing Australia currently approved safety vests are shown in Figure 1.

Although the introduction of safety vests in 1998 and the use of the vests shown in Figure 1, spine and torso injuries are still commonly sustained by jockeys (66–73). Globally talking, the most common injuries to jockeys are fractures and soft tissue damage, but the most serious are head and spinal damage, which can cause permanent, debilitating injuries and even death (74–78). The catastrophic injuries often suffered by jockeys in the torso area following falls highlight the poor level of protection offered by safety vests nowadays in use (67, 79, 80).

The consequences of falls incurred by jockeys are overall still severe, as often reported by media only or used those data for medical papers as per aforementioned (81–85). Nonetheless, according to Turner et al. (86), the common denominator in epidemiological jockey falls and injury studies are usually the race starts. Racetrack surfaces play a huge role in the success of a horse race as each surface type may affect the pace and effort required by many horses. Australia has racetracks made of turf, sand, dirt, or the latest synthetic range (*Polytrack*) thus, each horse is suited to a various kind and a certain classification within that track surface category. Notably, *Polytrack* is used even in Singapore, France, UK. New South Wales and Queensland are the only two Australian states where the direction of racing is clockwise, all the other States race in an anticlockwise direction. Contrary, American races are run counterclockwise and show considerable variability across the country in terms of racetrack design and geometry, the nature of the track surface, and the length of the races (87). All of these factors contribute to the risk of injury, both fatal and non-fatal, but the variability across tracks makes it difficult to determine the relative importance of the different variables. Besides, changes in surface functional properties, such as shear strength and surface stiffness, will have an impact on track performance (88). Racetrack surfaces may have significant temperature changes in a day and that can be detrimental to equine musculoskeletal health and thus, considered a risk factor for both the animals and the jockeys. Hence, a safety vest should protect jockeys throughout their entire journey: from the starting gates, during the flat race, until they cross the finish line, and until they return to the jockeys' room. Track conditions should be analyzed and considered during the safety vests design production because of their impact on users' needs: a fact that is currently not taken into consideration during that stage. At to date, track conditions have been analyzed during epidemiological studies (89–92).

The major international industry that provides clothing and equipment for horse riders is an interesting case of what motivates product development because the market for PPE for equestrian sports and leisure activities sees the proportion of amateur riders vastly outnumber professional riders. Jockeys form a small niche group within the totality of riders who might need or want to wear a safety vest: their safety equipment needs to be especially light and also accommodate jockeys' very light build and sometimes small stature. According to Racing Australia (93), in 2021/22, Australia had 35,103 active horses (flat and jumps races) along with 811 riders (jockeys, apprentice jockeys, and amateur). The weight that horses carry in a race is limited to enable them to perform without being overly taxed. Particularly, jockeys work hard to keep their own body weight as light as possible, ideally between 49 and 54 kgs, while they ride horses that weigh between 500 and 600 kgs. Specifically, Australian races ranged between 1,000 and 3,200 m and accordingly the standards weight for age for flat races may vary between 43.5 to 59.5 Kg: however, there are special considerations for the Melbourne and Caulfield Cups (63). The contrast between jockeys and thoroughbred horses is significant, like David and Goliath, and it represents another pivotal factor to take into serious consideration during the development of their PPE, particularly safety vests.

### 2.2. The most popular injuries in horse riding

**Figure 2:**
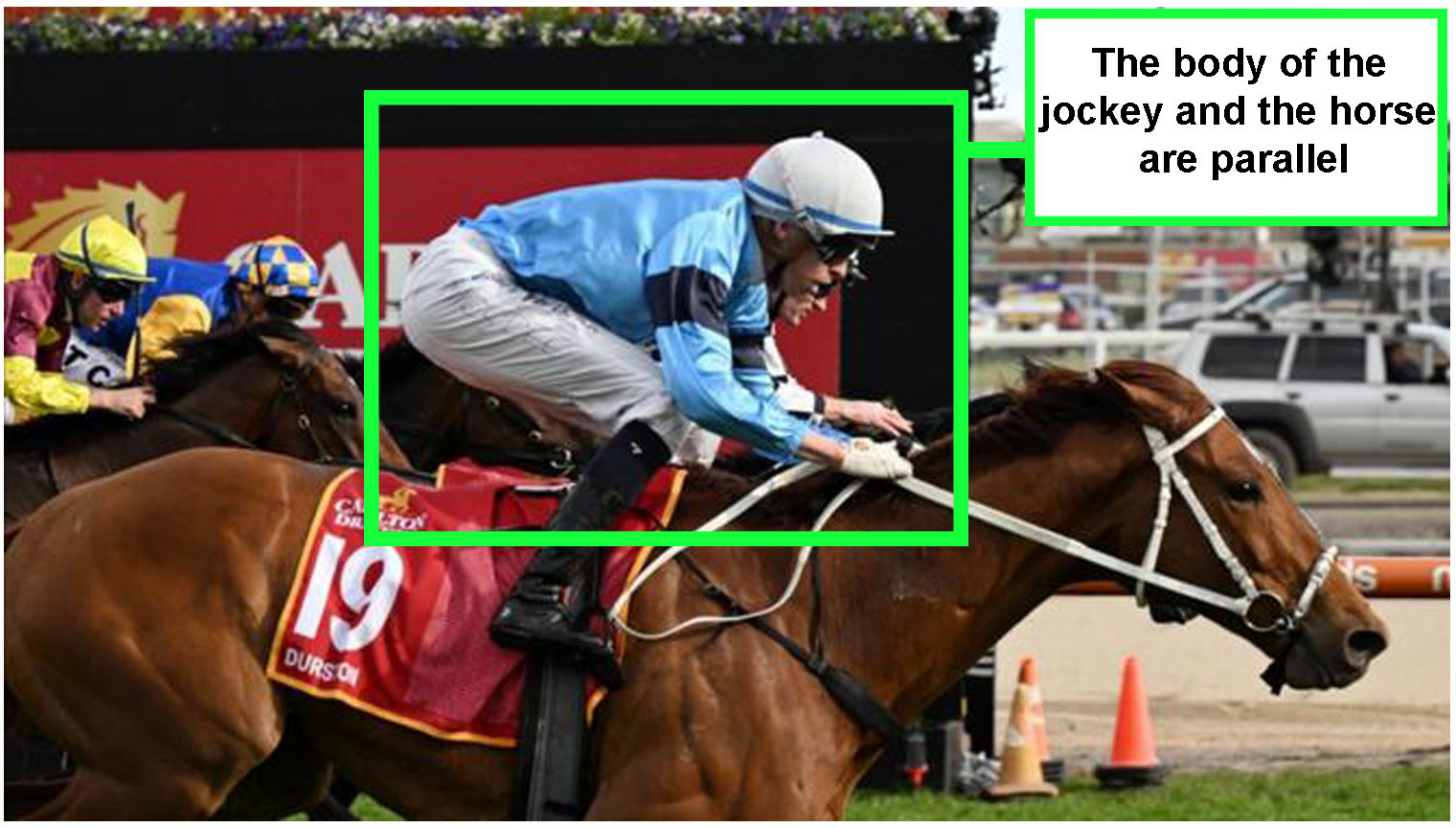
Martini glass: jockeys precarious riding posture [photo (94):].

Being a jockey carries a substantial risk of injury and even death. Although rates of injury in Australia are not exceptional by international standards, there may be an improvement in safety standards and PPE in the Australian racing industry. Jockeys face many challenges, and one is their precarious riding position, known among the industry as the “Martini Glass” (refer to Figure 2): a posture where their center of gravity sits over the horse's shoulders to minimize the effect of the jockey's weight on the horse's forward momentum. To achieve this, jockeys ride with very short stirrups attached to a minuscule saddle and they ride horses galloping in a group at a speed of around 60 Km/h (47). To reach and maintain the racing posture, jockeys target their training on the core, lower body, and legs (95). To control the horse, upper body training and extremely strong balancing skills are needed: thus, jockeys often also train on a simulator to sense the movements of a race (25, 96–98). Their racing posture has become an international success: therefore, this sees jockeys perched on top of the horse in a crouch position with only their lower legs in contact with the horse's body, surely exposed to a higher risk of injuries in case of falls and also impacts how they wear safety vests (99).

When an incident occurs, jockeys are extremely vulnerable to falls and it is pivotal to have their body extremely flexible thus, the front part of their safety vests should be short enough to allow jockeys to ride in the crouched posture but still capable of protecting their ribs and the other vital organs (100). In addition, multiple horses can fall at one time, exposing jockeys to the risk of being crushed or trampled by their own horse or those following (101, 102). Particularly, the epidemiological study conducted in Ireland by O'Connor et al. (75) states that professional horse racing is a popular but risky sport around the world. In Ireland, the rate of injuries in flat racing during the period 2011–2015 was 352.8 per 1,000 falls. As a consequence of these falls, the most popular injuries reported by jockeys were soft tissue (61.54%), fractures (15.38%), lower limb (32.89%) and concussion. These types of injuries are often experienced by Australian jockeys too: particularly, those who ride in flat races experience an average of one fall every 240 rides, with a third of falls resulting in injury (26, 103, 104).

Due to the risks that jockeys face, doctors and paramedics attend all race meetings to monitor the well-being of riders and as even per the literature, horse-related injuries provide a major public health concern throughout the world (105–107). Those most frequently involved in horse-related accidents are young females and the literature states that they are most commonly involved in falls from horseback, which more likely happen during warm days when the horses behave in a further unexpected manner (71). The kind of injuries and their location differs by the primary mechanism of injury, but many times involve body regions where the head and upper extremities are, along with common fractures and soft tissue injuries. Once a fatality occurs, neurological traumas are often experienced by jockeys (108–111): however, some improvements in horse related accident numbers and outcomes have been observed with the introduction of PPE such as helmets and safety vests. PPE is pivotal in preserving athletes' safety when participating in sports: these are of diverse nature, and the PPE literature often debates ways in which design innovation and the advanced materials along with wearable sensors available have been utilized to safeguard athletes from injury. Although there is no specific literature discussing the design of jockey's safety vests and the possibility of “converting” them into wearable technology, wearing appropriate and properly fitted protective equipment, clothing, and footwear (the aim is to be protected from head to toe) helps in preventing around 50% of sports injuries (18).

Besides, the weather conditions may influence the horses' behavior: on windy days, a racehorse can be frightened by the bangs and rattles from built structures and signage around the racetrack (112, 113). Instead, poor weather conditions can influence the communication utilized by jockeys during a race to avoid accidents caused by horses getting too close to each other. Thus, race riding involves a mix of adrenaline, the rush of speed and the thirst for victory. It does not matter if a jockey is racing on a country track or at a major city race meeting, getting across the line first is all that matters in the final stages of a horse race. Each victory pushes jockeys to return to the saddle day after day, risking their lives each time: as a matter of fact, jockeys must face consequences in case of falls. Turner et al. (86) state that jockeys, according to the injuries reported to racecourse medical professionals in France, Ireland, and the UK over the period 1992–2001, have an “incidence of 1–2 injuries per 1,000 rides in flat racing and 6–12 injuries per 1,000 rides in jump racing” (p. 704). Jockeys are aware that they are exposed every day to high risk, knowing that at some point in their career, they are likely to fall and get hurt, but they cannot predict how bad their injuries will be (50).

Notwithstanding the reduction in the number of jockeys' deaths since the introduction of the compulsory use of safety vests, their effectiveness has come under sporadic criticisms: Roe et al. (76) asked for the efficacy of these products to be evaluated alongside a safety education program being introduced for all horse riders. Equally, Foote et al. (114) confirmed that wearing safety vests and helmets during race riding is important, but they raised doubts about the validity of the product standards for PPE for jockeys. Their report analyzed the injuries and risk factors suffered by jockeys and a paucity of data about the incidence and type of injuries sustained by jockeys in thoroughbred racing was found. While confirming the importance of wearing safety vests and helmets, Foote et al. (114) criticized the variety of standards covering PPE for jockeys. Specifically, safety vests for Australian jockeys nowadays must comply with ARB Standard 1.1998 or European Standard EN 13158, while jockey's helmets must comply with AS/NZS 3838 2006; EN 1384:2012 or EN 1384:2017; ASTM F1163:20-04:Rev A 2011, ASTM F1163:20-13 or ASTM F1163:20-15; BS PAS 015:2011; VG1 01.040, Recommendation for Use, 12/12/2014 (also referred to as VG1 01.040: 2014–12). According to Racing Australia (93), all helmets must be fitted with “a nylon interlocking chinstrap clip attachment” (p. 63–64).

Despite safety vests are one of the main PPE worn by jockeys while with horses, the design methodology that provides a solution-based approach to solving problems has been poorly applied to their development, creating a need to understand better the nature and purpose of their use in order to establish the scope to improve their performance and wearability from the perspective of the whole user experience. In contrast, contemporary product design is seen as an integrative and iterative form of problem-solving that takes previous shortcomings into account, requiring a holistic understanding of the user and the context of use, and involving interdisciplinary collaboration with technical experts to develop the most innovative and effective concepts (115–117). Hence, the author drew on the conceptual framework of User Experience Design (UXD) research to place jockeys and medical professionals as users of safety vests to understand how their experiences and insights may inform the direction of what a new standard should take into consideration to address their needs and be a better guarantor of their safety.

### 2.3. Study design and data collection

As an early study in the field, the author has argued for the composition of the qualitative-research design based on its relevance to a UCD process grounded in understanding participants' experiences with and perceptions of safety vests. The application of a qualitative research method enabled the author to act as an instrument to gather the data required from a natural setting, but still as a way to obtain answers about the possible application of wearable technology in the jockeys' safety vests field. Through the application of qualitative research focusing on meaning in context, employing data gathering instruments that are sensitive to the hidden meaning, the author believes in her correct use of research design, which is based on constructivist epistemology and takes a qualitative, interpretive sense-making approach to guideline building.

Besides, she applied the case study methodology that is typically utilized to focus on a small group or situation to collect information about them on a specific topic: but because research design acknowledges that a researcher's subjectivity may lead to biases, the author utilized multiple data-gathering methods aiming to an acceptance of trustworthiness and validity in the case of data obtained from diverse participants, which were comparable and substantial (118). Choosing a qualitative-research method led her in beginning with a desk research project to summarize the history and evolution of safety vest design both from academic and industry perspectives. Specifically, this helped in clarifying the users' needs and also established the leading insights from the Australian horse-racing industry: this stage then supported the author with the participants' conversations because, as Sottsass (119) believed that design has complex relationships that must maintain if it wishes to be culturally effective thus, she holistically approached the users to gain their personal insights on these safety gear.

The data collection began with individual semi-structured interviews (which particularly happened between July and November 2016), which aimed to identify the users' needs and thoughts. Despite standardized questions being used across interviews, the author referred to prompts to ensure that the specific points to be covered were addressed but the participants had the flexibility to make unstructured contributions thanks to the semi-structured interview method chosen. Two kinds of prompt questions were used due to the different natures of the participants (please refer to Supplementary Appendix S1 and S2) and just as a protocol to develop the interviews. With this method, the author had the opportunity of exploring the participants' beliefs, insights, moral codes, experiences, and knowledge about the research topic. After the completion of semi-structured interviews, she conducted a preliminary analysis of the data obtained with the assistance of *NVivo* software to test the trustworthiness of the interview results: these preliminary findings informed the conduct of a focus group, which was her next activity (120, 121).

In October 2016, the focus group was conducted to familiarly discuss the issues experienced by the participants with their safety vests: both the participants and the author had known each other already due to the previous data gathering stages and this helped in creating a comfortable atmosphere for both parties to voice their ideas (122). Obtaining a deep understanding of the design context and the users' experiences with the safety vests was her main purpose because discovering what users really need and want is core in design research while she established a deeper engagement with users' emotional attachments to their belongings via the observation. During this phase (specifically, from July 2016 to July 2017), the author had the opportunity of watching the participants in their normal environments around the track, in the jockeys' room, the press room, the main community room and the weights room. More observation was performed at Victoria's Apprentices School and at the fitness sessions at Exercise Research Australia (ERA), where she was able to record this stage with notes and some photographs, in which participants' faces have been never shown.

### 2.4. Participants

The author focused on those jockeys that were primarily based in Melbourne (Victoria, Australia), riding flat races in the metro area only, as well as on local medical professionals. Overall, the group of participants was comprised of apprentice jockeys (*n* = 6), fully qualified jockeys (*n* = 9), and former jockeys (*n* = 2): significantly, 16 of the 17 participant jockeys had experienced at least one fall during their career when wearing safety vests. In addition to jockeys, two doctors and one intensive care paramedic participates due to these professionals having significant experience in treating jockeys' injuries (for more details, please refer to Table 1).

**Table 1:** The participants: codes, racing, and falls experiences.

Code	Participant category	Gender	Years of experience	Falls undergone	International races experience
AJ 01	Apprentice Jockey	M	5	Yes	No
AJ 02	Apprentice Jockey	F	4	No	No
AJ 03	Apprentice Jockey	M	2	Yes	No
AJ 04	Apprentice Jockey	M	3	Yes	No
AJ 05	Apprentice Jockey	M	3	Yes	No
J 01	Jockey	F	6	Yes	No
J 02	Jockey	M	21	Yes	Yes
J 03	Jockey	M	24	Yes	Yes
J 04	Jockey	F	28	Yes	Yes
J 05	Jockey	M	30	Yes	Yes
J 06	Jockey	M	18	Yes	Yes
J 07	Jockey	F	5	Yes	Yes
J 08	Jockey	F	18	Yes	Yes
D 01	Doctor	M	28	N/A	N/A
ICP	Intensive Care Paramedic	M	3	N/A	N/A
D 02	Doctor	M	10	N/A	N/A
AJ 06	Apprentice Jockey	M	3	Yes	No
J 09	Jockey	M	16	Yes	Yes
JR 01	Retired Jockey	M	28	Yes	Yes
JR 02	Retired Jockey	M	12	Yes	Yes

All the participants shown in Table 1 reported English as their first language, and they were aged 18 plus, all gender welcomed. Another important criterion to be satisfied was that no participants were diagnosed with anxiety, depression, or any other emotional disturbance in the 12 months prior to the data collection phase. Thanks to these criteria, the author was able to meticulously select the most suitable participants who met the scope of this case study besides, their privacy has always been preserved by using codes rather than names. Specifically, the jockeys participants could be distinguished into the less-experienced horse riders, those showing a more neutral response to the design of current safety vests, and into the older and more experienced jockeys, which were more reluctant in appreciating it because have ridden prior and after that safety vests became mandatory along with both national and international riding experiences.

### 2.5. Data analysis

The data collected during the interview, focus group, and observation phases were recorded using an audio recorder along with the author taking notes. The focus was on producing an accurate representation of participants’ thoughts and responses examined, which were combined during analysis, to build a picture of users' needs for jockeys' safety vests. This kind of analysis may group data into a consistent topic, and it was conducted with the *NVivo* software aid tool to identify and collate discrepancies in the data and prepare those for thematic analysis. The employ of *NVivo* software supported the author in organizing and breaking down the interviews and focus group data by assigning codes to words and phrases within the data: these codes were actively identified by the author as having meaning concerning the main research topic. Collecting data as audio recordings offered the benefit that their retention as recorded documents enabled access to them at any time to reconstruct detailed transcripts. The author was able to examine the recordings by listening to small parts at a time and also repeating the most significant and interesting parts to gain a better understanding. Consequently, semi-structured interviews and focus group data were divided into audio and text kinds. The audio data were then transcribed into text: interview questions and transcriptions were reviewed to obtain a general sense of the data gathered. The text was analyzed as a proxy for experience, while the transcripts as text were scanned using thematic analysis.

The subjectivity of ethnographic methods and the nature of such case study research often brought to think that biases introduced by the researcher during the data collection and its analysis may occur. Because each research method intrinsically has certain inbuilt biases and perspectives, the author utilized multiple methods and sources of data to reduce possible bias to construct a more holistic, objective, and credible picture of a case study of primary and dependent-secondary users in practice. Hence, by involving diverse participants and including multiple methods of data collection, the biases and uncertainties of convenience sampling were significantly reduced. In inductive empirical research based on the observation or experience of both the research subjects and the researcher, data triangulation is an important addition to the research design to produce robust research findings, even where research results are convergent, inconsistent, and contradictory (123–125). In social sciences, the concept of triangulation has a long history and is described as the mixing of data or methods where different angles or standpoints focus on a topic (126, 127). Moreover, triangulation, as a mixing data types, may support in validating the claims that can arise from an initial feasibility study and for this reason, is still capturing the interest of quantitative, qualitative, and mixed methods scholars alike. Thus, the author applied multiple methods of data collection, which established rigorous findings because the data triangulation reinforced the validity and the growth of utility.

Jockeys were peculiarly enthusiastic about sharing their experiences and thoughts with a researcher about their falls, injuries, and discomfort while wearing the safety vests, as well as their perceptions of the limitations of these products. Therefore, one of the participating jockeys, J02, clarified that:

*“For what we do there is always no guarantee …. And again, the perception is not that we expect the vest to save our lives …. All we want it to do is to help us, not hinder us in a racing incident”*

Although all the participants merged in indicating that the mandatory use of safety vests brought protective benefits, they even converged in saying that some issues were introduced with it as well, especially about body restrictions of movement, lack of protection in areas such as the spine and abdomen, and a paucity of innovations mainly in terms of design and materials, which also affected the use of ergonomics. Limited movements are a serious problem for jockeys, who need to who need to bend their heads, turn to look around for other runners, talk to each other during races, and be able to roll into a ball in the case of a nosedive fall, which is a common form of tumble. Most of the jockeys participating in this study described the vests as uncomfortable or rigid, body restrictions experienced during races, and often hot and heavy to wear. Usually, jockeys are slight and of small stature, but they differ from each other based on height, weight, gender, and age: these characteristics need serious consideration when designing safety vests due to still unsatisfied users. The majority of participants commented and discussed the aforementioned issues, particularly described by J06: “I think, some vests, they can be very restrictive with their (in) flexibility … It's just a little bit too rigid. Anything that's too rigid on you, I find it probably detrimental to your safety”.

It is a fact that female jockeys have different necessities from males due to their body shapes and having differentiated safety vests may help in addressing these requirements, as requested by AJ02:

*“From a female's point of view or perspective, I think definitely they need to have a male and a female vest. That's my opinion. They do it with all the motorbike gear and all that sort of stuff. The only reason I know is because I used to ride a lot of dirt bikes when I was younger. There's a big difference with the female body suits compared to the male's body suit. Obviously, we’ve got our breasts and our hips and stuff—our curves. It was all fitted; it was completely different. I just think, as a female, they definitely need to work on that a little bit. Just to make it as little bit more comfortable for us. Also, you want that light vest, but you also want the stability in it as well and the comfort, material-wise, too. So, it's probably saying the impossible, really …”*

Along with that, female jockeys often commented that the safety vests worn during track work, which are not covered by ARB 1.1998, can be more comfortable than those required for racing because softer but heavier. This increases their comfort because the vests tend to mold to the female body shape more easily. With the safety vests worn during racing seen as a source of discomfort, several participants reported wearing the safety vests differently from how they were meant to be worn, for instance, leaving the vest a bit looser on the sides or even wearing them backwards. In this case, they are even more exposed to risks of injuries.

Many of the participants highlighted the issues they encountered as the vests came into contact with their helmets while riding horses due to the particular riding posture: this highlighted the question of the interaction of standards because most of the helmet manufacturers have no connection with those producing the safety vests. Consequently, a paucity of research into the conditions of use has created this risky situation during races: the top of the safety vest's back may bump into the back of a jockey's helmet. This indicates the need to introduce a correct use of ergonomics to enable female and younger jockeys to feel greater comfort in wearing the safety vest, since comfort affected jockeys' focus and thus, is core for their safety. Participating jockeys clearly explained that the vests interfered with their race helmets, leading to bigger issues such as their vision being impaired while riding, as J06 described below:

*“I am not watching where I am going because I have to look with my eyes up instead of my head up … I can feel it pinching on the back of the vest, so it is just half an inch, so it is stopping me from extending my neck forward. Jockeys cannot extend forward properly because vest and helmet bump together.”*

This situation becomes most critical during the last 400 meters of a race, when jockeys particularly urge their horses up to their maximum speed to try to cut the finish line first and win the race, with a pack of horses jostling each other to be the winner. Those jockeys with more years of experience surely were the most critical as they had encountered the issues with the vest for a longer time and without seeing anything changing. Some of them started to ride prior that the safety vests becoming compulsory thus, they had even experiences in horse riding with and without the vests. Thus, J04 simply stated “I don't know how they do the standards” while J03 commented “I am not convinced the standards are right” instead, J02 voiced their perception of the source of the problem by stating:

*“The people doing the test might be engineers and experts in testing equipment, but they are not experts in riding, racing, or dealing with the animals or what we deal with. They are only dealing with numbers, facts and obviously video footage, but they are not the people actually riding, or the ones actually falling in it.”*

Simultaneously, medical professionals reinforced jockeys' concerns by highlighting the nature and severity of injuries treated and how the safety vests impact their actions. Particularly, they reported the crucial actions they do following a fall because the time a jockey spends lying on the turf is pivotal. It's when medical professionals need to act quickly and accurately. Unfortunately, the vests' design represents an impediment to this rapid supply of aid due to being hard to remove from injured or unconscious jockeys. At the same time, the participating medicals shared their curiosity about this research process since finally, someone was trying to investigate such an important safety matter, with the scope to bring improvements to how they carry out their work. Discussions, mainly with those jockeys that had experienced falls and the medical professionals, were arisen even about the pieces utilized and the methods of assembly of the safety vests may be problems. Specifically, the participant ICP shared that:

*“A better-functioning, better-performing vest will be good and provide better protection, easy to use, comfortable for the jockeys and easy removal by other people, because you have to remember that, oftentimes, we are trying to remove the vests and the jockeys are lying on the ground and often we have to roll them so they’re flat on their back …So, the weight of their bodies is lying on the vests and so it can be very hard to get them off.”*

In the specific circumstances of a fall, both jockeys and medical staff considered the most dangerous place for it to happen the starting barriers or at full gallop while perched above the saddle. Particularly, the medical participants believe that the safety vest designs were not exactly fit for purpose because, during a fall, a jockey's chin often comes forward and caught in the front top of the vest thus, adding to a jockey's injuries. The vests' design was described by 75% of participants as not exactly right for their needs: in addition, once a fall occurs, a jockey's chin often comes forward and catches in the top of the vest prior to causing bruises and cuts, as D02 shared:

*“The injuries vary a lot, and any part of the body can be injured, from simple things like soft-tissue injuries and bruising, through to fractures, minor and major fractures, head injury, chest injury, abdominal injury …I have seen injuries in all parts of the body.”*

Throughout the interviews, participants discussed the positives and negatives that they had experienced due to the wearing of safety vests. Overall, the whole group of participants accepted the compulsory use of the vests because they acknowledged that the vests offered some improvement in their protection. However, 60% of jockeys, still feared that the use of the safety vests might be linked to the number of spinal injuries currently registered. Remarkably, none of the participants in this study felt uncomfortable when talking about such a sensitive topic with the author and nobody requested to leave. During the entire data collection phase, the author experienced a professional and friendly atmosphere due to the choice of safe and comfortable environments for both parties.

## 3. Results

### 3.1. Field research and observation

Such are the constraining effects of the Australian product standards that the participants were definitely surprised that they were being asked about their experience with safety vests. Particularly, jockeys demonstrated consciousness about how risky their profession can be and even how they welcome the PPE products on the market even if not totally guarantee their safety: they had often tried to provide feedback on them but feel like have been ignored.

All the participating jockeys deeply and precisely shared their feeling of restriction when wearing the vests, attributing this to a system that had not paid attention to their concerns or not involved them in the development of the standards for vest design (128). Experience limited movement is a pivotal issue for jockeys: they need to bend their head, turn to look around for other horses, talk to each other during races, and be able to roll into a ball if a nosedive fall occurs (a common form of tumble where jockeys may be flung forward into the ground). Of course, even their “Martini glass” riding posture has an impact on how they might fall and how they desire to wear safety vests. Jockeys are still seeking safety, comfort, and PPE lighting weight: core characteristics for their activities.

### 3.2. Results and insights

Overall, jockeys considered safety vests as compulsory items rather than essential and desired personal protective equipment to guarantee their safety. Hence, to begin balancing safety and comfort in the provision of safety vests, there are two options: (1) to use the current technology (e.g., wearable sensors) to improve an existing product design or (2) to employ a radical approach. Consequently, each of these choices has its advantages and disadvantages (129). An incremental innovation approach has the advantage that the product remains competitive, and acceptance of any change is easier in being delivered via an already recognizable product. Revisions to an existing design can be implemented at a reasonable price and if successful can be marketed to a large market of recreational and competitive horse riders in addition to jockeys. The design of Australian safety vests for jockeys sees opportunities for incremental improvement spanning addressing the basic and rigid design, the lack of ergonomics, the paucity of advanced materials and technologies, the spinal area not being adequately protected, the absence of a user-centered design approach to enhance marketability, providing alternative design for male and female jockeys, and facilitate and assist with the medical professionals' aid job.

To talk about inclusive design, the users' needs must be satisfied through a product that can please multiple and diverse users. Both UXD and UCD focus on providing products that develop and obtain a great experience with users: along with a radical innovation approach, fresh opportunities for wholly new products and markets may be created, opening doors to new innovative PPE companies to enter the marketplace. Concepts that should be applied to the whole PPE sports categories, especially in the development of safety vests for jockeys. In this case, radical product innovation can be the only answer to most of the deficits that jockeys attribute to safety vests as their designs enabled by the current standards may be too compromised to be sufficiently adapted. Still, inspired by the use of the product ecology framework, a pivotal consideration in the opportunity for radical evolution in this field is in approaching them not only as personal protective pieces of equipment but also as first aid devices for treating jockeys' injuries and facilitating the medical staff's job.

Ergonomics represents the missing point in the design of safety vests: it is pivotal to address both male and female jockeys' needs and even transform this protective gear into wearable technology, offering benefits for the medical staff too. However, the standards represent a problem to address those needs and bring innovation: 30% of jockeys participating in this study confirmed concerns and provided negative feedback about the systems for drawing up the standards and the consequence of it for users. The remaining 70% offered the same thoughts but in a more informal way, still providing personal experiences and intimate insights about this topic. Overall, the majority of participants highlighted their concerns and doubts about the safety of the neck and spine of the wearer: this is one of the most sensitive topics in the horse racing industry still. The participants provided sufficient evidence to suggest that a lack of ergonomics is a basic factor that negatively influenced the jockeys' experience of the safety vests.

Poor ergonomics is shown to have affected at least two key aspects of contemporary racing safety: the satisfaction of female jockeys with the vests and the input by track-side health professionals to the evolution of vest designs. Track-side health professionals are currently involved in injury recording in the Australian Racing Incident/Injury Database: information on the safety vests used should be recorded on these forms. Additionally, the Single National System should have a record of each jockey's safety vests and accordingly, of the safety equipment used thus, this can be used as denominator data at each race ride and used as the basis for further research. Further, the standards currently in use in Australia were perceived as suppressing the application of modern contemporary materials and health-based sensor technology to these products. Consequently, this research found that both ergonomics and the input of track-side medical professionals remained excluded from jockey vest designs and standards.

Thus, enhancing a safety vest with wearable technology leads to achieving a balance between interaction design, technology, purpose, and comfort. The application of wearable sensors and/or smart materials may help in quickly identifying the jockeys' injury, understanding its location and severity of impact in a fall or contact between the horse and jockeys, that can be stored in a dedicated database and easily passed among the medical professionals in services at the racetracks. Besides, such information can be sent ahead to emergency department staff at hospitals if necessary while waiting to receive injured jockeys.

## 4. Discussion and implications

This study acknowledges that being a jockey is a unique and hazardous occupation; hence, personal protective equipment such as safety vests should help to save jockeys' lives. Research has demonstrated the importance of wearing protective equipment during any activities involving horses, yet a review of the literature demonstrates that these products are lacking in scientific research specifically connected to jockeys' safety factors. Although there is a broad range of literature related to horse riding, much of it is tangential rather than specific to the research topic. Thus, the author began with the following main research question: *What are the relevant primary and dependent-user factors that, in themselves and in combination with each other, should substantially influence product innovation in the case of safety vests for jockeys?* However, she found it useful to break it into the following sub-queries to aid analysis: *What are the main historical and regulatory factors that influence the design of jockeys' safety vests? Why is a UCD method not yet applied to enhance the effectiveness of safety vests for jockeys, and which may offer benefits? Is it possible to integrate fast-changing technology and advanced materials into safety vests?*

In answering those interrelated research questions set out, she examined the evolution of safety vest design in the Australian horse racing industry by identifying the case study of primary and dependent-secondary users affecting the evolution of this product's design. This study also confirmed that, in Australia, safety vests are yet inadequate for the task of preventing or minimizing jockeys' injuries. Jockeys’ safety vests belong to the PPE category in a niche but high-risk sport: jockeys' injuries share similarities with those experiences by other athletes in high-impact sports, like hockey, boxing, sky, or football. As this paper has argued, neither incremental nor radical product innovation is currently possible in Australia due to the constraining effects of the product standards: the ARB 1.1998 created on request of the Australian Rules Board, shows criteria that have been only minimally and rarely reviewed over the past 25 years. Thus, jockeys' perceptions and experiences led them to share in-depth doubts with the author about the knowledge of those entitled to establishing and maintaining the safety vests standards. The most relevant findings to be used as the basis for future research are summarized in Table 2.

**Table 2:** Summary of the key recommendations for further research.

Main Findings	Main topics to conduct further research	Guidelines
Users are not involved in the design development of safety vests for jockeys.	Product Design	Users can significantly contribute to the creation of a prototype, with particular tips on its design. The neck cut of both the front and back of the vests needs revisions.
Users must be able to roll in case of falls and without hindrance, yet the current vests are bulky and hampered this specific need.		
Users’ needs are not satisfied by the current safety vests.		
The EN 13158:2009 still applied in Australia is considered too generic because mainly considers all horse riders rather than jockeys.	Product Safety Standards	Users should be involved in the revision and/or creation of a new standard, which focuses on jockeys’ safety vests and allows the use of the latest materials and technologies. A wider audience should be then considered.
The standards applied in Australia represent an obstacle to the use of new materials and technologies (which are needed to satisfy users’ needs).		
The standards applied in Australia did not take into consideration the use of ergonomics (which would help in improving wearability for females or with particularly skinny and/or particular stature jockeys).		
The standards applied in Australia are different to those stipulated for helmets, and this lack of cohesion creates interference between the two products for the user because often helmets and safety vests bumped together during the race.		

Surely, the introduction of a new design may offer a higher level of safety and comfort; besides, a revision of the neck cut of both the front and back of safety vests represents a scenario in which the standards would be reinterpreted to develop related standards also for jockeys' vest and helmet design. Medical professionals would receive benefits from product innovation in ways such as the reduction of time to removing the vests from injured jockeys or having the possibility to store those injuries in a dedicated database and identify the injury locations in a quicker time. Along with that, there is a dearth of alignment between standards for products that interact during application as well as the challenge of adapting international standards to reflect local conditions (e.g., weather, turf states). Achieving this outcome involves ongoing research for new designs to be introduced to the market, especially because of the constant innovation in materials, digital, and wearable technologies.

Wearable technologies are currently making strong inroads into sports, being associated with enhanced functionality and design. According to Seshadri et al. (6), the data gained from wearable sensors offer a vast toolkit for many professionals (e.g., athletes trainers, physicians and physiotherapists, sports scientists) to make real-time decisions. Many sports and competitions (like the Olympics) have high standards and coaches and athletes constantly seek any possible advantage (e.g., equipment innovations, lighter materials to wear, dietary regiments, novel training tools and technologies) to maximize their performance and enhance the chances of success (130). In the everyday life, we are now able to measure and track various aspects of our health and performance thanks to various devices, like Fitbit, Apple watch, Garmin or Polar (131, 132). Any wearable technology has the capacity of being useful to keep track of athletes when training grounds were shut down because of the COVID-19 pandemic (133, 134). Specifically, the English Premier League took advantages of keep tracking their players during remote training due to the country lockdown (135–137). Because wearable technology refers to anything attached to the human body with the aim of measuring aspects of performance while practicing a physical activity like running, swimming, or walking: thus, it may be useful to extend further research in the safety vests for jockeys to take advantage like other sports already did for the athletes' benefits.

### 4.1. The crucial role of product design to satisfy users’ needs

The interdisciplinary field of producing products by reverse engineering nature, better known as *biomimetics* or *biomimicry*, supports researchers study natural phenomena to gain ideas from nature and apply them to solve real world human issues. While design thinking emphasizes starting the design process with a focus on empathy for human users, biomimicry extends that concept to include all life forms. Consequently, the author for instance considered spiders for this study because they can obtain a durable fabric design instead, while chameleons may be helpful in creating fabrics able to communicate the injury's location to medical professionals. Chameleons' skin is covered with several layers of special cells named chromatophores, which respond to chemicals from the nervous system and bloodstream.

**Figure 3:**
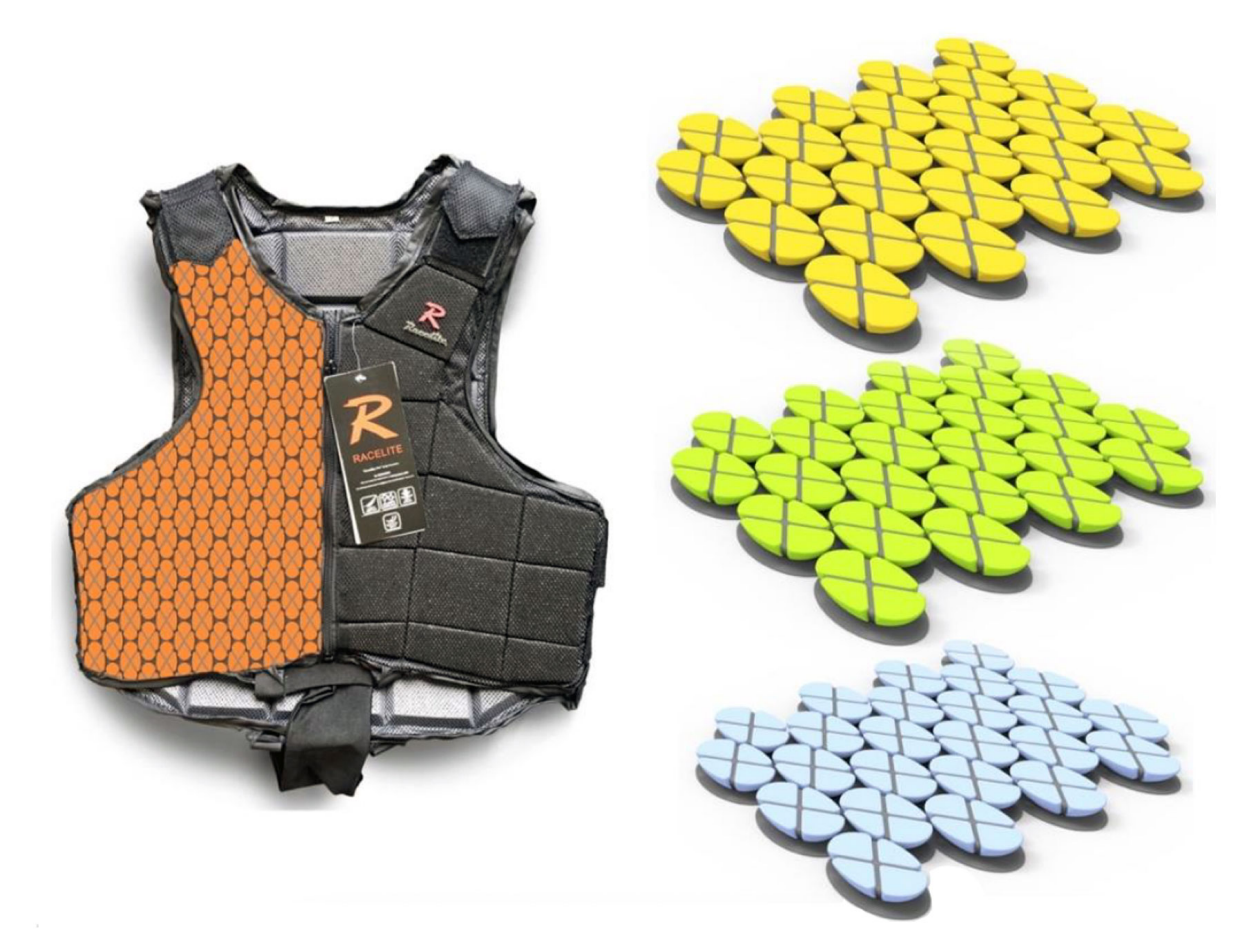
Proposed pattern variations.

The author suggests more research for the application of a new super-thin fabric composite material, made up of strips that reflect various wavelengths of light, which may convert safety vests into wearable technologies. Further research may investigate the possibility of applying fabric that as the chameleons' skin when bends or moves reveal strips that reflect various wavelengths and effectively change color. Each cell can be expanded from an invisible dot into a colorful disk, that offers color to a corresponding portion of the skin, which is layered. In this way, safety vests might communicate the jockeys' injuries: hence, the author considered spiders and flowers to obtain a durable fabric with a suitable design, while the camouflage of chameleons may be referred to as a support in creating materials able to quickly (e.g., changing colors) communicate the injury's location to the medical professionals. Figure 3 shows a proposed new pattern shape, in which two colors are utilized to explain the use of a combination of materials for diverse functions.

Besides, via the application of advanced material/s along with wearable sensors, a dedicated database with jockeys’ medical conditions and injuries experienced may be possible: this will be available for the authorized medical professional when needed. With just a baby step like the simple introduction of a variation in the design padding of the jockeys' safety vests, the author showed that despite no differences in the standards, an improvement of wearability and flexibility to accomplish users' needs may be possible. Besides, the proposed design may have wearable sensors integrated to support the medical staff in collecting jockeys' injury data or to assess the injury's severity via the material's color changing, data that can be received and stored on their phones too. Instead, to minimize or solve the problem experienced by the medical professionals in removing the vests if needed, the author found relevant the possibility of embroidering small and light-weight magnets into these safety products. More research and prototyping in this field are strongly recommended.

This study showed that there are safety products and users that are less trendy than others, and there are even more, but they still need to see ergonomics applied in their field. In addition, challenging the *status quo* and the product standards may allow designers to consider multiple users and apply the latest materials and technologies to create innovative products (65). Being able in transforming safety vests into wearable technology may lead to the input of ergonomics applications, provide better protection to jockeys, and facilitate the health professionals' services. To summarize, research findings indicate that design-led innovation is the linchpin for primary and dependent-secondary users' design, specifically for jockeys' safety vests. Users seek safety while performing in a career they love, but the standards represent an obstacle to the meeting of users’ needs. This is demonstrated in the paucity of ergonomics that results in a lack of attention, particularly to female jockeys' needs, and the limit that the standards produce regarding the use of advanced materials and technologies such as sensors, and application of innovation to the vest template. Besides, the role of industrial design is growing, and the researcher's role to seek new processes that inform the design of future developments will also grow, to meet the final users' needs. The link between research and industrial design enjoys mutual benefits to produce positive achievements, even in this research field. Significantly, these findings may be beneficial to other high-impact sports (e.g., skiing, hockey, boxing, or motorcycling) where users experience similar injuries to jockeys and horse riders too. Further research in this field and the development of prototypes are highly recommended.

## 5. Limitations

The small sample size and the latest technologies and smart materials available that may have occurred naturally over time should be taken into consideration. Larger-scale participants and a safety vest prototype are needed to further develop these findings.

## 6. Conclusion

The employment of analytics in sports medicine when applying data from wearable technology is having a great clinical impact when monitoring and forecasting the workload and cardiovascular health of the athletes, along with their sleep quality tracking, and assessing their hydrating and weight status to prevent related injuries. However, there are situations where these advantages are still merely applied, such as regarding the jockeys' safety vests. This is a field that clearly needs innovation to enhance the function and experience of these safety products. Besides, the number of female jockeys is constantly growing, and this indicates the necessity of offering customized products that take into consideration gender, age, and body shape to address users' needs while still keeping them safe.

This qualitative study has argued that research-informed design should include jockeys and medical professionals as the core, not only to deliver improved vest designs but even to serve as a catalyst for the revision of safety vests' standards in ways that are dynamic and context-specific. The wealthy Australian thoroughbred industry has many users who interact with safety vests instead, horse riders from show jumping, eventing, and pony clubs may be potential further users of safety vests as wearable tech. Therefore, an innovative product can be quickly and positively accepted by the market. Horse riding is a dangerous occupation, yet safety vests are still inadequate to prevent severe injuries to jockeys, which endure a high injury rate due to the nature of their profession. To conclude, the implementation of wearable sensors to enhance the protection offered by jockeys' safety vests may be the quickest answer in supporting these athletes and in designing new PPE. Constant monitoring of jockeys' injuries and training should be mandatory and collected in a database for each state.

## Data Availability

The original contributions presented in the study are included in the article/**Supplementary Material**, further inquiries can be directed to the corresponding author.
