# Supplementary material for: Wearable technology may assist in reducing jockeys' injuries if integrated into their safety vests: a qualitative study
Source: Front Sports Act Living. 2023 Jun 21;5:1167110. doi: 10.3389/fspor.2023.1167110 (PMC10321524; doi:10.3389/fspor.2023.1167110)
Supplement: Supplementary file 1 [file Datasheet1.docx]

**TABLE 1:** The participants: codes, racing, and falls experiences

| **Code** | **Participant category** | **Gender** | **Years of experience** | **Falls undergone** | **International races experience** |
| --- | --- | --- | --- | --- | --- |
| AJ 01 | Apprentice Jockey | M | 5 | Yes | No |
| AJ 02 | Apprentice Jockey | F | 4 | No | No |
| AJ 03 | Apprentice Jockey | M | 2 | Yes | No |
| AJ 04 | Apprentice Jockey | M | 3 | Yes | No |
| AJ 05 | Apprentice Jockey | M | 3 | Yes | No |
| J 01 | Jockey | F | 6 | Yes | No |
| J 02 | Jockey | M | 21 | Yes | Yes |
| J 03 | Jockey | M | 24 | Yes | Yes |
| J 04 | Jockey | F | 28 | Yes | Yes |
| J 05 | Jockey | M | 30 | Yes | Yes |
| J 06 | Jockey | M | 18 | Yes | Yes |
| J 07 | Jockey | F | 5 | Yes | Yes |
| J 08 | Jockey | F | 18 | Yes | Yes |
| D 01 | Doctor | M | 28 | N/A | N/A |
| ICP | Intensive Care Paramedic | M | 3 | N/A | N/A |
| D 02 | Doctor | M | 10 | N/A | N/A |
| AJ 06 | Apprentice Jockey | M | 3 | Yes | No |
| J 09 | Jockey | M | 16 | Yes | Yes |
| JR 01 | Retired Jockey | M | 28 | Yes | Yes |
| JR 02 | Retired Jockey | M | 12 | Yes | Yes |
