# Supplementary material for: Wearable technology may assist in reducing jockeys' injuries if integrated into their safety vests: a qualitative study
Source: Front Sports Act Living. 2023 Jun 21;5:1167110. doi: 10.3389/fspor.2023.1167110 (PMC10321524; doi:10.3389/fspor.2023.1167110)
Supplement: Supplementary file 2 [file Datasheet2.docx]

# Appendix II - Prompt questions for semi-structured interviews (Medical Staff)

The interview protocol developed for the medical professionals participating in this study involved the following questions to begin the conversation:

1. Can you describe how and when you started to provide professional medical help to jockeys?
2. What are the most common injuries you have treated as a consequence of a fall? Please describe this in detail.
3. What do you think are the strengths and benefits of wearing a safety vest?
4. What are the main functions and benefits that you expect from a safety vest?
5. What is the main function that you expect from a safety vest that could contribute to your medical services?
6. What could the redesign of a safety vest do to improve the medical staff services at racecourses in case of a fall?
7. What should a safety vest look like to you?
8. Would like to be involved in a development of a safety vest prototype?
